# Towards routine monitoring for myocardial injury after noncardiac surgery

**DOI:** 10.1186/s13741-026-00660-x

**Published:** 2026-03-25

**Authors:** Dushanthi Dissanayake, Su-Yin MacDonell, P. J. Devereaux, Janny Xue Chen Ke

**Affiliations:** 1 Department of Anesthesia, Pain Management & Perioperative Medicine, Dalhousie University, Halifax, Canada; 2 Department of Anesthesiology, Pharmacology & Therapeutics, Faculty of Medicine, The University of British Columbia, Vancouver, BC Canada; 3 Department of Anesthesia, St. Paul’s Hospital/Providence Health Care, Vancouver, BC Canada; 4 Department of Health Evidence and Impact and Department of Medicine, McMaster University, Hamilton, Canada; 5 Population Health Research Institute and World Health Research Institute, Hamilton, Canada

**Keywords:** Myocardial injury after noncardiac surgery, Perioperative medicine, Implementation, Troponin

## Abstract

Myocardial injury after noncardiac surgery (MINS) occurs in approximately 13% of adults ≥ 45 years of age who undergo major inpatient noncardiac surgery and is associated with an increased risk of 30-day and 1-year mortality. Routine perioperative troponin monitoring is needed to avoid missing the majority of MINS events. Although there are challenges to the implementation of MINS surveillance, there has been enormous growth in the uptake of MINS monitoring across Canadian centres. In this paper, we explore challenges to routine screening for MINS and strategies to overcome them. The key challenges were stratifying who to monitor, management strategies and outcomes requiring further research, and resource limitations. Several groups have documented high uptake of perioperative troponin monitoring at centres that have established institutional protocols. Strategies to facilitate implementation of the MINS guidelines include multidisciplinary collaboration, standardized clinical pathways, and audit and feedback. Implementation of a routine MINS monitoring program requires concerted multidisciplinary effort throughout the perioperative period, encompassing the preadmission clinic, postoperative wards, and outpatient clinics.

## Background

The concept of Myocardial injury after noncardiac surgery (MINS) was first published in 2014. Since this time, there has been exponential growth in articles on this topic, culminating in guidelines and scientific statements. MINS occurs in approximately 13% of adults ≥ 45 years of age who undergo major inpatient noncardiac surgery and is associated with an increased risk of 30-day and 1-year mortality (Vascular Events in Noncardiac Surgery Patients Cohort Evaluation (VISION) Study Investigators et al., 2019). MINS is defined as an elevated postoperative cardiac troponin level secondary to an ischemic etiology, with or without ischemic symptoms or ECG changes, occurring within 30 days of non-cardiac surgery (Ruetzler et al. 2021). Ninety percent of MINS events occur by postoperative day two (Ruetzler et al. 2021). As 84 to 93% of MINS events are asymptomatic (Ruetzler et al. 2021; Duceppe et al. 2017), systematic monitoring is crucial. Guidelines for risk stratification and troponin monitoring have been developed by societies including the Canadian Cardiovascular Society (CCS), the American Heart Association (AHA), and the European Society of Cardiology (ESC) (Duceppe et al. 2017; Thompson et al., 2024; Halvorsen et al. 2022) (eAppendix Table 3) to monitor for MINS.

Although there are challenges to the implementation of MINS surveillance, there has been enormous growth in the uptake of MINS monitoring across Canadian centres (Dale-Gandar et al. 2024). Among the 24 departments that responded to a survey of 99 Canadian anesthesiology departments, 67% (16/24) routinely performed preoperative risk stratification and 54% (13/24) routinely monitored postoperative troponin in accordance with the CCS guidelines (Dale-Gandar et al. 2024). The most frequently reported barriers for guideline implementation included insufficient facility space for postoperative monitoring (42%, 10/24), lack of departmental protocol (37%, 9/24), limited availability of existing staff (33%, 8/24), limitations in department engagement (29%, 7/24), and limited support/engagement from other specialties (25%, 6/24) (Dale-Gandar et al. 2024). Amongst patients who meet eligibility criteria from society guidelines for perioperative biomarker monitoring, completion of different components of the MINS monitoring protocol varied by institution and population. For example, testing rates ranged from 9% for preoperative N-terminal pro-B-type natriuretic peptide (NT-proBNP) risk stratification for postoperative troponin monitoring (Gouda et al. 2021) to 84% for preoperative and postoperative troponin monitoring (Stahlschmidt et al. 2023) (Table 1). Implementation of a routine MINS monitoring program requires concerted multidisciplinary effort throughout the perioperative period, encompassing the preadmission clinic, postoperative wards, and outpatient clinics. In this paper, we explore challenges to routine screening for MINS and strategies to overcome them.

**Table 1:** Barriers and recommendations for monitoring for myocardial injury after noncardiac surgery (MINS) from institutions with established routine MINS monitoring programs

Article	Location	Inclusion Criteria	Guidelines followed	MINS Monitoring Strategy	Rate of Implementation	Definition and management of MINS	Perceived Barriers	Recommendations
Liem 2018 (Liem et al. 2018)	Erasmus University Medical Centre, Rotterdam, Netherlands	Patients undergoing intermediate- or high-risk non-cardiac surgery with an expected postoperative stay ≥ 24 h, and aged ≥ 60 years	2014 ESC/ESA	HS Troponin T on POD 1 to 3, unless discharged earlier, or when clinically indicated	73.3% (3538/4825) of eligible patients received troponin testing	HS Troponin T ≥ 50 ng/L. Management strategy not reported	Lack of electronic laboratory orders	Not reported
McIsaac 2021 (McIsaac et al. 2021)	Ottawa Hospital General Campus, Ottawa, Ontario	All patients undergoing elective, non-cardiac surgery who met the following criteria: 1) anticipated postoperative, and 2) age ≥ 65 or age 45–64 with a RCRI ≥ 1	2016 CCS	Preoperative troponin Troponin I within 6 h postoperatively. Troponin I measurements on POD 1 to 3, or until day of discharge (whichever occurred first) Routine ECG within 6 h postoperatively	74.1% (530/715) of patients received at least one troponin test post-implementation 84.4% (54/64) of patients with an elevated troponin measurement received appropriate follow-up care	For Troponin I > 45 ng/L: Inpatient consults (internal medicine [troponin I 45 to 1000 ng/L], cardiology [troponin I > 1000 ng/L]), nuclear stress myocardial perfusion imaging within one week of discharge, and follow-up with cardiologist or internist within two weeks of discharge	Timing of internal medicine review (e.g., "Evening/night consults will probably be pushed to the morning due to lack of urgency to see these patients”) Concerns about ability to accommodate follow-up at the outpatient internal medicine clinic Concerns about how to manage MINS patients Concerns about increasing workload (e.g., increased consult burden.)	Multidisciplinary engagement with key stakeholders (e.g., cardiologists, internists, surgeons, nurses, anesthesiologists, residents, and clinical research staff) in the form of semi-structured interviews to identify and address barriers to implementation Design more efficient screening forms Optimize workflow to minimize burden on clinical staff Dissemination of information by perioperative leadership to prepare staff and detail upcoming changes
Pelland 2020 (Pelland et al. 2020)	St. Paul’s Hospital, Vancouver, Canada	Surgical patients who met the criteria for MINS protocol activation based on the 2016 CCS guidelines	2016 CCS	Preoperative NT-proBNP Postoperative ECG and troponin in PACU Daily postoperative troponin for 3 days	After implementing the bundled interventions: Protocol activation for eligible patients: 81.3% (339/417) Protocol completion for eligible patients: 43.1% (79/183)	Internal medicine consult	Not reported	Bundled interventions (preoperative, intraoperative, and postoperative protocols) Raise awareness of the 2016 CCS guidelines (e.g., at grand rounds) Development of an electronic pre-printed order set for MINS monitoring Integration of care between perioperative anesthesiology and surgical teams Provide data on departmental protocol adherence rates to improve compliance
Popova 2023 Popova et al. 2023)	Hospital de la Santa Creu i Sant Pau, Barcelona, Spain	Patients aged ≥ 65 or patients aged < 65 with at least one documented antecedent of cardiovascular disease (e.g., coronary artery disease, chronic heart failure, stroke, transient ischemic attack, peripheral vascular disease) or impaired renal function (eGFR < 60) undergoing elective or urgent noncardiac surgery requiring overnight hospital admission	2014 ESC	Preoperative HS troponin (at the preoperative visit or just before surgery) Postoperative HS troponin at 48 and 72 h after surgery	63.3% (1477/2333) of eligible patients completed the implementation study	HS troponin ≥ 14 ng/L and an increase of ≥ 50% from the preoperative value: cardiology evaluation	“Personnel unavailability” on weekend and holiday surgeries (resulted in a loss of 24.3% (568/2333) of eligible cases) Lack of automatic requests for blood sampling, cardiology evaluations, and follow-up reminders	Multidisciplinary team to enact the protocol Increase awareness, acceptance, and understanding among healthcare professionals about the importance of MINS monitoring Increase availability of necessary resources and infrastructure (e.g., IT support to enable automatic requests for necessary tests, sufficient personnel to conduct timely assessments of all eligible patients) Develop an easily understandable monitoring protocol Dedicated team to facilitate protocol compliance
Puelacher 2018 (Puelacher et al. 2018)	University Hospital Basel, Switzerland	Patients undergoing noncardiac surgery with an expected postoperative length of hospitalization ≥ 24 h, and were aged ≥ 65 or aged ≥ 45 with a history of coronary artery disease, peripheral artery disease, or stroke who were undergoing inpatient noncardiac surgery	2014 ESC	Preoperative HS troponin within 30 days before surgery Postoperative troponin on POD 1 and POD 2, and later if clinically indicated	Not reported	HS troponin ≥ 14 ng/L above preoperative values (or between 2 postoperative values if the preoperative value was missing) within 7 days of surgery: cardiology consultation triggered electronically	Not reported	Implement a routine screening program Clinicians automatically alerted of eligibility for routine monitoring based on the electronic health records
Stahlschmidt 2023 (Stahlschmidt et al. 2023)	Hospital de Clínicas de Porto Alegre, Porto Alegre, Brazil	Patients ≥ 16 years of age undergoing high-risk (predicted 30-day mortality ≥ 5% according to the Ex-Care risk model) non-cardiac surgery with a planned hospital length of stay ≥ two nights	HS troponin was measured in all patients deemed as “high-risk” based on the Ex-Care model	Preoperative HS troponin T Postoperative HS troponin T 24 and 48 h	84% (442/525) of eligible patients had “at least one HS troponin T.”	HS troponin T > 65 ng/L or absolute change 40 ng/L: cardiology consultation	Overly broad definition of the target population for serial troponin measurement (based on VISION (1)) Lack of knowledge about the underlying mechanisms of perioperative myocardial injury Lack of consensus for standardised treatment of MINS	Employ a validated risk stratification tool to standardise risk stratification Bundle postoperative surveillance interventions for high-risk surgical patients (e.g., in this study: 1) preoperative risk identification and communication, 2) adoption of a high-risk PACU discharge checklist, 3) prompt nursing admission to ward, 4) intensification of vital signs monitoring, 5) prompt access to medical support, and 6) troponin measurement)
van Waes 2013 (Waes et al. 2013)	University Medical Center Utrecht, Utrecht, Netherlands	Patients aged ≥ 60 undergoing intermediate- to high-risk noncardiac surgery under general or spinal anesthesia, and had an expected postoperative length of hospital stay of ≥ 24 h	No established guideline was followed	Third-generation troponin I routinely measured in eligible patients for POD 1 to 3	73.4% (1627/2216) of eligible patients received at least one troponin measurement 40.9% (907/2216) of eligible patients received troponin measurements on all three postoperative days	Troponin I > 0.06 µg/L(lowest value measurable with a 10% coefficient of variation above the 99th percentile of assay): further diagnostic procedures, including ECG or cardiology consultation, per ward physician discretion	Not reported	Not reported
Xi 2024 (Xi et al. 2024)	Second Medical Center, Chinese PLA General Hospital	Patients who were aged ≥ 65 or aged ≥ 50 with a history of cardiovascular disease or cardiovascular risk factors (e.g., smoking, obesity, hypertension, diabetes, and dyslipidemia) who underwent intermediate- or high-risk non-cardiac surgery (according to the ESC/ESA surgical risk score (Halvorsen et al. 2022))	2022 ESC	For intermediate- and high-risk noncardiac surgery: preoperative BNP, troponin T (within 14 days preoperatively), and 12-lead ECG. Postoperative troponin at 24 and 48 h	91% (561/613) of eligible patients received a preoperative BNP 97% (594/613) of eligible patients received a preoperative ECG 100% of patients received a preoperative troponin measurement 100% of patients received a troponin measurement within 3 days of surgery	Troponin T ≥ 30 ng/L within 3 days postoperatively. No specific treatment reported, though there is established surgeon and internist co-management	Significant burden to independently manage the required monitoring	Team-based approach to care with surgical and medical co-management

*Abbreviations*: *ASA* American Society of Anesthesiologists, *BNP* Brain natriuretic peptide, *CCS* Canadian Cardiovascular Society, *ECG* Electrocardiogram, *eGFR* estimated glomerular filtration rate, European Society of Anesthesiology *ESC* European Society of Cardiology, *HS* High-sensitivity, *MINS* Myocardial injury after noncardiac surgery, *NT-proBNP* N-terminal pro-B-type natriuretic peptide, *PACU* Post-anesthesia care unit, *POD* Post-operative day, *RCRI* Revised Cardiac Risk Index

### Who and how to monitor

MINS screening must balance close surveillance of patients who will likely develop MINS against unnecessary bloodwork and resource allocation in low-risk patients. Although there are variations across guidelines regarding which patients should have perioperative troponin monitoring, there are commonly accepted patient groups across guidelines (e.g., patients age ≥ 65 years or patients with known cardiovascular disease) (eAppendix Table 3) (Duceppe et al. 2017; Thompson et al., 2024; Halvorsen et al. 2022). Because most MINS events occur within 48 h and almost all within 72 h after surgery, guidelines recommend monitoring troponin values daily for 48 h after surgery. The CCS guidelines also recommended troponin monitoring on day 3 after surgery.

Research and implementation of MINS protocols have evolved as troponin and BNP assays evolved, with variations amongst guidelines and institutions. In the American Heart Association MINS guidelines, MINS is diagnosed as “elevated postoperative cardiac troponin with ≥ 1 cardiac troponin measurement above the 99th percentile of the upper reference limit for the cardiac troponin assay, with a rise/fall pattern indicative of acute myocardial injury” occurring the first 30 days postoperatively, that is “attributable to a presumed ischemic mechanism …. in the absence of an overt precipitating nonischemic cause” (Ruetzler et al. 2021). Assay-specific prognostically important thresholds are “non–high-sensitivity fourth-generation troponin T ≥ 30 ng/L (Roche fourth-generation Elecsys troponin T assay), high-sensitivity troponin T of 20 to < 65 ng/L with an absolute change of ≥ 5 ng/L, or a high-sensitivity troponin T concentration ≥ 65 ng/L (Roche Elecsys high-sensitivity troponin T assay)” (Ruetzler et al. 2021). The AHA guidelines further specified thresholds among patients with abnormal baseline troponin values (Ruetzler et al. 2021). Further studies are required to understand the validity of extrapolating across older generation and high-sensitivity troponin assays. In the meantime, clinicians could consider using specific assay thresholds for assays used at their institution for the most evidence-based diagnosis and prognosis.

### Management of MINS

Current MINS management recommendations include further investigations for risk stratification, lifestyle modification (physical activity, diet modification, smoking cessation), optimization of cardiovascular risk factors, long-term aspirin and statin therapy, and dabigatran (Duceppe et al. 2017; Thompson et al., 2024; Halvorsen et al. 2022). An observational study of 5633 patients with MINS, of which 38% of these patients had a cardiology consult, demonstrated in propensity score matched analyses that cardiology consultation was associated with improved 30-day mortality (HR, 0.64; 95% CI, 0.50–0.81) and cardiovascular mortality (HR, 0.58; 95% CI, 0.35–0.95) (Oh et al. 2021). A survey of Canadian anesthesiology departments revealed that 46% (11/24) of centres had a dedicated multidisciplinary perioperative anesthesiology service, (Dale-Gandar et al. 2024) highlighting the need for further growth.

Observational studies, based on risk-adjusted analyses, suggest patients with MINS benefit from aspirin, statin, beta-blocker, and angiotensin converting enzyme inhibitor (ACEI) (Gouda et al. 2021; Devereaux et al. 2011). Currently, dabigatran is the only treatment for MINS supported by a randomized controlled trial, the Management of Myocardial Injury after Non-Cardiac Surgery (MANAGE) trial (Thompson et al., 2024). Because the CCS guidelines were published before MANAGE was reported, they do not have recommendations regarding perioperative dabigatran for patients with MINS. The ESC guidelines suggest considering dabigatran treatment 1 week after noncardiac surgery in patients who have MINS. The AHA guidelines state that “postoperative administration of DOACs may decrease the risk of long-term cardiovascular events” (Thompson et al., 2024). However, they also emphasize that further investigation is needed to confirm the role of postoperative antithrombotic therapy in patients with MINS (Thompson et al., 2024). The management of MINS remains an area of ongoing research (Ruetzler et al. 2021), and routine screening to identify MINS patients will help build the research infrastructure and clinical experiences to better understand MINS treatment and outcomes.

### Resource limitations

The cost-effectiveness of MINS monitoring and treatment may depend on the extent that they ultimately change patient outcomes. The latest CCS guidelines employed a cost-effective approach by recommending inexpensive biomarkers for preoperative risk stratification, rather than costly non-invasive cardiac tests (Duceppe et al. 2017). Buse et al. (2017) performed a model-based cost-consequence analysis of a perioperative troponin monitoring program amongst 6021 patients enrolled in Vascular Events in Noncardiac Surgery Patients Cohort Evaluation (VISION) (Buse et al. 2018). They found that compared to standard of care, troponin monitoring (four 4th generation troponin T measurements and further tests and consultations in patients with increased troponin T levels) incurred an incremental cost of $112 (2015 Canadian dollars) per screened patient (Buse et al. 2018). The incremental cost to avoid missing a MINS case was $1632 (2015 Canadian dollars), and monitoring for troponin was more cost-effectiveness in subgroups with increased risk of MINS (Buse et al. 2018). McIsaac et al., 2021) evaluated the financial impact of implementation of the CCS MINS monitoring guidelines and did not find differences in cost, length of stay, or complication rates (McIsaac et al. 2021). With regards to the financial implications of MINS treatment, Lamy et al., 2022) found that dabigatran treatment, as per the MANAGE trial, is cost neutral, since the cost of the drug was offset by savings from having fewer clinical events and procedures (Lamy et al. 2022).

### Strategies from existing MINS monitoring programs

Effective strategies toward guideline implementation in clinical practice include the use of care pathways, educational meetings, changes in organizational culture, and audit and feedback (Pereira et al. 2022) (Table 1 and 2). Specifically related to the implementation of MINS monitoring, eight established MINS programs (Table 1) have published strategies based on their experiences, which include multidisciplinary collaboration, standardized clinical pathways, and audit and feedback. Among these studies, there was a high proportion of patients who received perioperative troponin monitoring as recommended by guidelines.

**Table 2:** Barriers and recommendations for monitoring for myocardial injury after noncardiac surgery (MINS) monitoring from studies evaluating regional uptake of MINS monitoring

First Author (Reference)	Location	Rate of Implementation	Barriers	Recommendations
Azizi 2023 Azizi et al. 2021)	Ontario, Canada	13.1% (24,408/186,699) of eligible patients as per the CCS guidelines received postoperative troponin testing	Unclear if increased testing and detection of MINS will lead to better patient outcomes	Not reported
Gouda 2021 (Gouda et al. 2021)	Alberta, Canada	By the end of the study period (2017), 8.8% (911/10351) of eligible patients across urban and other sites underwent preoperative natriuretic peptide screening Of patients with an elevated preoperative natriuretic peptide, 40.9% (274/670) received postoperative troponin monitoring	Limited data to guide MINS treatment Restricted access to natriuretic peptide testing in certain parts of Alberta Failure to identify high-risk patients preoperatively Inadequate provider understanding of MINS (asymptomatic patients, unclear impact on outcomes)	Automated preoperative cardiac screening to prompt perioperative biomarker testing as recommended by the CCS guidelines Physician education regarding the importance of MINS monitoring
Dale-Gandar 2024 (Dale-Gandar et al. 2024)	Canada	Of 99 university-affiliated Canadian anesthesia departments surveyed, 24.2% (24/99) responded 66.7% (16/24) reported that their hospital routinely performed preoperative risk stratification 54.2% (13/24) routinely used postoperative troponin surveillance in line with CCS guidelines	Insufficient facility space for postoperative monitoring Lack of a departmental protocol Limited availability of existing staff Limited departmental engagement Limited support/engagement from other specialties Cost of tests required Limited departmental awareness Limited departmental engagement Perceived inability to change outcome Renumeration of additional staff to perform preoperative and postoperative surveillance	Provide education and support for guideline adoption, such as strategies from centres with successful implementation Establish a dedicated team of perioperative physicians and a local champion to increase engagement and guideline adoption

*Abbreviations*: *BNP* Brain natriuretic peptide, *NT-proBNP* N-terminal pro-B-type natriuretic peptide, *MINS* Myocardial injury after noncardiac surgery, *CCS* Canadian Cardiovascular Society

The implementation of a MINS monitoring program requires sustained collaboration amongst perioperative care stakeholders, ranging from anesthesiologists, internists, cardiologists, surgeons, nurses, laboratory medicine teams, to patients (McIsaac et al. 2021; Fischer et al. 2016). Engagement with stakeholders enables identification of barriers to implementation, and the development of strategies to overcome these barriers, increasing the likelihood of guideline adoption (McIsaac et al. 2021; Fischer et al. 2016). Integration of care amongst anesthesiology, surgical, and medicine teams would be essential to facilitate guideline-directed care (McIsaac et al. 2021; Pelland et al. 2020). To integrate care seamlessly, there must be effective communication amongst anesthesiology, surgical, and medicine teams about MINS protocol activation, including proper documentation (Pelland et al. 2020). Clear roles should be defined within the perioperative team and tailored to the institution; as is already done in many institutions, anesthesiologists could monitor for MINS and refer to internal medicine or cardiology services when MINS has been detected. To facilitate buy-in to MINS protocols, sustained efforts to increase awareness and acceptance of the guidelines by perioperative health professionals are crucial (Fischer et al. 2016; Pelland et al. 2020; Popova et al. 2023).

Secondly, standardized clinical pathways, such electronic health record order sets and reminders, can support guideline implementation, increase efficiency, and minimize provider variability (Pelland et al. 2020; Popova et al. 2023; Puelacher et al. 2018; Liem et al. 2018). To support MINS monitoring at St. Paul’s Hospital (Vancouver, BC, Canada), Pelland et al*.* 2020) developed order sets that facilitated protocol completion, and appropriate postoperative monitoring (Pelland et al. 2020). Reminders to prompt healthcare professionals to perform guideline-directed assessment and care may also be beneficial for increasing compliance (Gouda et al. 2021; Fischer et al. 2016). For example, automated reminders can be incorporated in the electronic health record or in patients’ physical charts to prompt healthcare professionals to assess patient eligibility for MINS monitoring and order appropriate preoperative and postoperative testing (Gouda et al. 2021; Fischer et al. 2016; Popova et al. 2023).

Lastly, to understand and incentivize ongoing guideline adherence, a program must be in place to regularly track and review protocol adherence rates (Fischer et al. 2016; Pelland et al. 2020). This can be achieved by having providers document protocol completion in the electronic health record and performing periodic chart reviews. Continuous reassessment of the MINS program will provide valuable feedback on the implementation process and help identify obstacles to adherence. Institutions can then collaborate with providers to develop strategies to overcome these obstacles, thereby enhancing compliance and patient care.

## Conclusion

In summary, MINS is associated with substantial mortality, and routine perioperative troponin monitoring is needed to avoid missing the majority of MINS events. The key challenges were stratifying who to monitor, management strategies and outcomes requiring further research, and resource limitations. Several groups have documented high uptake of perioperative troponin monitoring at centres that have established institutional protocols. Strategies to facilitate implementation of the MINS guidelines include multidisciplinary collaboration, standardized clinical pathways, and audit and feedback. Multidisciplinary collaborations across perioperative care teams are needed to develop pathways for routine screening and management for MINS.

## Data Availability

No datasets were generated or analysed during the current study.

## Appendix

**Table:** Appendix Table 3 MINS monitoring guidelines with their corresponding recommendations

	**Guideline**		
	**Canadian Cardiovascular Society Guideline** Duceppe (2017)	**American Heart Association Guideline** Thompson (2024)	**European Society of Cardiology Guideline ** Halvorsen (2022)
Eligibility criteria for MINS monitoring	Patients with an elevated preoperative NT-proBNP/BNP or, if the preoperative NT-proBNP/BNP measurement is unavailable, patients who have an RCRI score ≥1, age 45-64 years with significant CVD, or age ≥ 65 years.	Patients with known CVD, symptoms of CVD, or age ≥65 years with CV risk factors undergoing elevated-risk NCS	Patients undergoing intermediate to high-risk NCS who are age ≥65 years, have known CVD, CV risk factors, or symptoms suggestive of CVD.
Postoperative Monitoring	Daily cTn for 48 to 72 hours and ECG in PACU. Consider in-hospital shared-care management.	Consider postoperative troponin surveillance by measuring cTn at 24 and 48 hours after surgery.	High-sensitivity cTn 24 and 48 hours after surgery.

*Abbreviations:**MINS* Myocardial injury after non-cardiac surgery, *CVD* Cardiovascular disease, *CV* Cardiovascular, *NCS* Noncardiac surgery, *cTn* cardiac troponin, *RCRI* Revised Cardiovascular Risk Index, *MACE* Major adverse cardiovascular event, *CABG* Coronary artery bypass graft, *ICD* Implantable cardioverter defibrillator, *BNP* Brain natriuretic peptide, *NT-proBNP* N-terminal pro-B-type natriuretic peptide, *ECG* Electrocardiogram, *CCTA* Coronary computed tomography angiography, *PACU* Post-anesthesia care unit
